# Cytokine inflammatory threat, but not LPS one, shortens GABAergic synaptic currents in the mouse spinal cord organotypic cultures

**DOI:** 10.1186/s12974-019-1519-z

**Published:** 2019-06-25

**Authors:** Vincenzo Giacco, Giulia Panattoni, Manuela Medelin, Elena Bonechi, Alessandra Aldinucci, Clara Ballerini, Laura Ballerini

**Affiliations:** 1 International School for Advanced Studies (SISSA/ISAS), 34136 Trieste, Italy; 2 Department of Life Sciences, University of Trieste, 34127 Trieste, Italy; 3 Department NEUROFARBA, University of Florence, 50139 Florence, Italy; 4 Dipartimento di Medicina Sperimentale e Clinica, University of Florence, 50139 Florence, Italy; 5 Present address: Wolfson Centre for Age Related Disease, King’s College London, Guy’s Campus, London, SE1 1UL UK

**Keywords:** Organotypic spinal slices, Patch-clamp, Synaptic currents, Neuroinflammation, GABAergic inhibition, GABAergic receptors, Spinal circuits, NKCC1, Resident neuroglia

## Abstract

**Background:**

Synaptic dysfunction, named synaptopathy, due to inflammatory status of the central nervous system (CNS) is a recognized factor potentially underlying both motor and cognitive dysfunctions in neurodegenerative diseases. To gain knowledge on the mechanistic interplay between local inflammation and synapse changes, we compared two diverse inflammatory paradigms, a cytokine cocktail (CKs; IL-1β, TNF-α, and GM-CSF) and LPS, and their ability to tune GABAergic current duration in spinal cord cultured circuits.

**Methods:**

We exploit spinal organotypic cultures, single-cell electrophysiology, immunocytochemistry, and confocal microscopy to explore synaptic currents and resident neuroglia reactivity upon CK or LPS incubation.

**Results:**

Local inflammation in slice cultures induced by CK or LPS stimulations boosts network activity; however, only CKs specifically reduced GABAergic current duration. We pharmacologically investigated the contribution of GABA_A_R α-subunits and suggested that a switch of GABA_A_R α1-subunit might have induced faster GABA_A_R decay time, weakening the inhibitory transmission.

**Conclusions:**

Lower GABAergic current duration could contribute to providing an aberrant excitatory transmission critical for pre-motor circuit tasks and represent a specific feature of a CK cocktail able to mimic an inflammatory reaction that spreads in the CNS. Our results describe a selective mechanism that could be triggered during specific inflammatory stress.

**Electronic supplementary material:**

The online version of this article (10.1186/s12974-019-1519-z) contains supplementary material, which is available to authorized users.

## Background

Neuroinflammation is a characterizing trait of various central nervous system (CNS) pathologies, from neurodegenerative diseases to neuropsychiatric disorders [1]. Currently, intense research efforts are dedicated to the understanding of how the different signaling pathways, activated in neurons and neuroglia by the inflammatory milieus, may ultimately promote synaptic dysfunction [2–4]. For example, several studies documented, in the interplay between the immune system and neuronal function, the involvement of pro-inflammatory cytokines (CKs), such as TNF-α, IL-1β, and IL-6 [5–7]. Similarly, lipopolysaccharide (LPS, an endotoxin derived from gram-negative bacteria)-induced neuroinflammation may lead to synaptic dysfunctional signaling contributing significantly to cognitive disturbances [8]. In contrast to the general agreement on the emergence of synaptopathy due to inflammation [2], the rules governing the specific neurotransmission systems involved, and their tuning, are unclear. Conflicting evidence indicate that exogenous CK applications may increase [9] or decrease [10] glutamatergic synaptic transmission, as well as for GABAergic transmission, where both decreases [11, 12] and increases [13, 14] are reported.

With the aim of dissecting the impact of immune status alterations on neural circuit function, we focused our study on the effects of local inflammation in a controlled micro-environment where neurons and neuroglial cells maintain appropriate organization: the organotypic slice cultures developed from the embryonic mouse spinal cord. In this complex in vitro model, the sensory-motor cytoarchitecture, synaptic properties, and spinal cord resident cells are retained in a 3D-fashion [15–17]. By the use of this model, we preliminarily reported the emergence of synaptopathy in pre-motor circuits following CK transient exposure, characterized by a speeding up of the decay phase of GABAergic inhibitory currents [17]. A broader question is to what extent the tuning of GABAergic current duration may occur as a response to any local alteration in the inflammatory status of the spinal cord, disrupting physiological excitability levels, being GABAergic neurotransmission an important determinant of spinal circuit coordination [18].

In the present study, we compare the effects on synaptic transmission of different experimental inflammatory models, LPS, a potent trigger of cytokine induction [19, 20] and the most common stimulus used to investigate microglial reactivity in brain inflammation, and a pro-inflammatory cocktail containing interleukin-1β (IL-1β), well-known determinant of neuropathy [1, 2], tumor necrosis factor α (TNF-α), present during Th1/Th17-mediated inflammatory reactions, and granulocyte macrophage-colony stimulating factor (GM-CSF), targeting resident microglial cells. These cytokines are key factors known to affect neuronal functions and responsible for pro-inflammatory effects in the CNS of multiple sclerosis animal models [21]. We adopted relatively acute treatments (hours), known to trigger inflammatory responses, without inducing direct neurotoxicity, yet still able to alter synaptic transmission [17]. Thus, organotypic spinal cord cultures were transiently exposed to pro-inflammatory CK cocktail (TNF-α, IL-1β, and GM-CSF, 4 and 6 h; [17]) or to LPS (4, 6, and 24 h).

To compare CKs and LPS impact on synaptic activity, in particular on GABAergic currents, we used single-cell patch-clamp recordings. We further explore by immunofluorescence and confocal microscopy resident neuroglia reactivity, and we measure the local production of cytokines and chemokines in response to the two inflammatory stresses. CKs and LPS significantly increase the generation of these signaling proteins, and both danger signals boost basal synaptic activity, inducing a distinct transformation of resident neuroglia morphology. To note, only CKs promote changes in inhibitory transmission time course. Finally, we investigated the mechanisms responsible for the shortening of GABAergic current duration upon CK treatment, since these may emerge as potential targets for novel therapeutics.

## Methods

### Organotypic spinal cord cultures, pro-inflammatory treatments, and pharmacology

All experiments were performed in accordance with the EU guidelines (2010/63/UE) and Italian law (Decree 26/14) and were approved by the local authority veterinary service and by our institution (SISSA) ethical committee. All efforts were made to minimize animal suffering and to reduce the number of animals used. Animal use was approved by the Italian Ministry of Health, in agreement with the EU Recommendation 2007/526/CE.

Organotypic spinal cord and dorsal root ganglia (DRG) slices were obtained from mouse embryos (C57BL/6 J) at E12-13 of gestation as previously described [15–17, 22]. Briefly, pregnant mice were sacrificed by CO_2_ overdose and fetuses delivered by cesarean section. Isolated fetuses were decapitated, and their backs were isolated from low thoracic and high lumbar regions and transversely sliced (275 μm) with a tissue chopper. After dissecting the spinal cord slices and the DRG from the surrounding tissue, slices were embedded into a thick matrix obtained by chicken plasma (Rockland) and thrombin (Sigma) clot. Slices were cultured in plastic tubes with 1 mL medium. The tubes were kept in a roller drum rotating 120 times per hour in an incubator at 37 °C in the presence of humidified atmosphere, with 5% CO_2_. Experiments were performed on spinal cultures at 14–21 days in vitro (DIV). The day of the experiment, organotypic slices were incubated with standard medium (control) or, for 4 or 6 h (4H and 6H), with two different inflammatory paradigms: (i) a cocktail of the mouse recombinant cytokines (10 ng/mL each) TNF-α (R&D Systems, #210-TA/CF), IL-1β (R&D Systems, #M15330), and granulocyte-macrophage colony-stimulating factor (GM-CSF; R&D Systems, #P04141; [17, 23]); (ii) lipopolysaccharide (LPS; 1 μg/mL, Sigma, O55:B5). For LPS, we also tested a longer incubation time point (24H). CKs or LPS were removed after the incubation times, prior to electrophysiological recordings.

Bumetanide (Sigma) was diluted in phosphate buffer solution (PBS 1×, Sigma) and used to block the Na^+^/K^+^/Cl^−^ co-transporter (NKCC1; [24]). To decrease the cytoplasmic chloride concentration, slices were incubated for 24 h at 37 °C with 10 μM bumetanide (BUM24H), after 24H, the CK cocktail was added for additional 4 h (BUM24H + CKs4H).

### Immunofluorescence, imaging, and analysis

Organotypic cultures were fixed with 4% formaldehyde (prepared from fresh paraformaldehyde; Sigma) in PBS (1×) for 1 h at room temperature (RT; 20 to 22 °C) and washed in PBS. Free aldehyde groups were quenched in 0.1 M glycine in PBS for 10 min. Slices were permeabilized and blocked in PBS 1×, 5% FBS (Sigma), 1% BSA (Sigma), and 0.3% Triton X-100 (Sigma) at RT for 1 h and then incubated overnight at 4 °C with anti-GFAP (mouse monoclonal, 1:400, Sigma), anti-Iba1 (rabbit polyclonal, 1:200, Wako), anti-SMI32 (mouse monoclonal, 1:200, EMD-Millipore), and anti-MAP 2 (mouse monoclonal, 1:200, Sigma) primary antibodies.

For β-tubulin III and GAD65/67 co-immuno-labeling, fixed samples were quenched with Na-(meta) periodate 2.3% in deionized water for 5 min and Na-borohydride 1% in TRIS 0.1 M for 10 min. Slices were blocked in free-floating with PBS 1×, 10% FBS (Sigma), 1% BSA (Sigma), 1% fish gelatin (Sigma), and 0.3% Triton X-100 (Sigma) at RT for 1 h and then incubated overnight at 4 °C with anti-β-tubulin III primary antibody (mouse monoclonal; 1:500, Sigma) and anti-GAD65/67 (rabbit polyclonal; 1:500, ABCAM).

Subsequently, the slices were PBS-washed and incubated with secondary antibodies diluted in blocking solution for 2 h at RT in the dark. The secondary antibodies were Alexa 488 goat anti-mouse (1:500, Invitrogen), Alexa 488 goat anti-rabbit (1:500, Invitrogen), Alexa 594 goat anti-mouse (1:500, Invitrogen), Alexa 594 goat anti-rabbit (1:500, Invitrogen), and DAPI (Thermo Fisher Scientific). Samples were mounted on glass coverslips using Vectashield mounting medium (Vector Laboratories).

Images were acquired using Nikon C2 confocal microscopes with Ar/Kr, He/NE, and UV laser with × 20, × 40, or × 63 oil objectives (1.4 N.A.) using oil mounting medium (1.515 refractive index). Confocal sections were acquired every 0.5 μm up to a total *Z*-stack thickness of 5 μm. For each condition, we performed > 3 and < 6 independent cultures; from each culture series, we used 4 slices, and from each slice, ≥ 5 fields were randomly acquired. Offline analysis of the image *Z*-stack was performed using the open source image-processing package FIJI (http://fiji.sc/Fiji).

For the quantitative analysis of microglia morphology, we used the particle measurement feature in *ImageJ*, to automatically measure the area and the perimeter, necessary to calculate the transformation index [25], as $\frac{{\left[\mathrm{perimeter}\ \mathrm{of}\ \mathrm{cell}\ \left(\upmu \mathrm{m}\right)\right]}^2}{4\uppi \bullet \left[\mathrm{area}\ \mathrm{of}\ \mathrm{cell}\ \left({\upmu \mathrm{m}}^2\right)\right]}$, which defines microglia ramification status. Indeed, cells with long processes and small soma show a large index that depends on cell shape, regardless of the cell size.

Quantification of GAD65/67 immunoreactivity was performed measuring the intensity of fluorescence and the number of GAD65/67 clusters using the Volocity3D Image Analysis Software. Clusters were determined after thresholding of images. Thresholds were determined using the “voxel spy” facility of the software and chosen such that all recognizable punctuate structures were included into the analysis (size > 0.03 μm^3^ and separate touching objects of 0.5 μm^3^).

### Electrophysiological recordings and data analysis

For patch-clamp recordings (whole-cell, voltage clamp mode), a coverslip with the spinal culture was positioned in a recording chamber, mounted on an inverted microscope (Nikon Eclipse TE200) and superfused with a standard saline solution containing (mM) 152 NaCl, 4 KCl, 1 MgCl2, 2 CaCl2, 10 HEPES, and 10 glucose; the pH was adjusted to 7.4 by NaOH (305 mOsm). Patch pipettes were pulled from borosilicate glass capillaries (4 to 7 MΩ) and filled with intracellular solution containing (mM) 120 K gluconate, 20 KCl, 10 HEPES, 10 EGTA, 2 MgCl_2_, and 2 Na_2_ATP. The pH was adjusted to 7.3 with KOH (295 mOsm). All electrophysiological recordings were performed at RT. The reported voltage values are corrected for the liquid junction potential (− 14 mV) [26]. Electrophysiological responses were amplified (EPC-7, HEKA), sampled, and digitized at 10 kHz with the pCLAMP software (Axon Instruments) for offline analysis. The value of series resistance was < 10 MΩ enabling recordings of synaptic currents without significant distortion and thus was not compensated for [16, 26]. Recordings were performed from ventrally located spinal interneurons visually identified based on previously reported criteria [27]. Spontaneous post-synaptic currents (PSCs) were recorded at − 70 mV holding potential by the Clampfit 10 software (pClamp suite, Axon Instruments). On average, ≥ 400 events were analyzed from each cell in order to obtain mean kinetic and amplitude parameters. From the average of these events, we measured the rise time defined as the 10–90% time needed to reach the peak of the synaptic current, the peak amplitude, and the decay time constant (τ) that was obtained by fitting a mono-exponential function [27].

We compared the passive membrane properties among control and CK- and LPS-treated spinal interneurons. We detected no differences in cell capacitance (51 ± 31 pF control; 43 ± 23 pF CKs 4H; 43 ± 21 pF CKs 6H; *n* = 62, 47, 54 respectively; 55 ± 23 pF control; 44 ± 18 pF LPS 4H; 48 ± 22 pF LPS 6H; *n* = 35, 38, 34, respectively) and input resistance (470 ± 195 MΩ control; 587 ± 137 MΩ CKs 4H; 481 ± 114 MΩ CKs 6H; 430 ± 123 MΩ control; 399 ± 121 MΩ LPS 4H; 439 ± 146 MΩ LPS 6H).

Inhibitory GABAergic post-synaptic currents (IPSCs) were recorded at − 84 mV holding potential in the presence of CNQX (10 μM; Sigma), strychnine (1 μM; Sigma), and APV (25 μM; Sigma). Tetrodotoxin (TTX; 1 μM, Latoxan) was used to isolate GABA_A_-receptor-mediated miniature events (mIPSCs).

Recordings of the IPSCs at different holding potentials were used to measure the chloride equilibrium potential (*E*_Cl_), which was determined as the *x*-axis intercept point of the resulting I/V curve extrapolated by linear regression.

The imidazopyridine zolpidem (zolpidem, Sigma), a benzodiazepine ligand with high selectivity for GABA_A_Rs containing the α1-subunit and moderate affinity to α2- or α3-subunit, [28], was dissolved in water stock solution (1 mM) and diluted to the concentration of 100 nM [29] in extracellular solution for bath application (15–20 min).

### Cytokine and chemokine measurement

TNF-α, IL-4, IL-6, IL-10, INF-γ, CXCL1, and CXCL2 concentrations were measured in organotypic culture supernatants by Milliplex assay (Merck Millipore, #MCYTOMAG-70 k), using the Bio-Plex apparatus (Biorad), according to the manufacturer’s recommendations.

### Statistical analysis

All values from samples subjected to the same experimental protocols were pooled together, and results are presented as mean ± S.D., if not stated otherwise, with *n* = number of neurons. A statistically significant difference between two data sets was assessed by Student’s *t* test (after checking variances homogeneity by Levene’s test) for parametric data and by Mann-Whitney’s test for non-parametric ones. Two-way analysis of variance (two-way ANOVA) and one-way ANOVA were used for parametric data or Kruskal-Wallis test for non-parametric ones, to determine significance when multiple groups were compared. Statistical significance was determined at *P* < 0.05.

In box plots, the thick horizontal bar indicates the median value, the cross indicates the mean value, the boxed area extends from the 25th to 75th percentiles while whiskers from the 5th to the 95th percentiles.

## Results

### CKs but not LPS regulate GABA_A_ receptor-mediated synaptic currents in spinal organotypic cultures

**Fig. 1:**
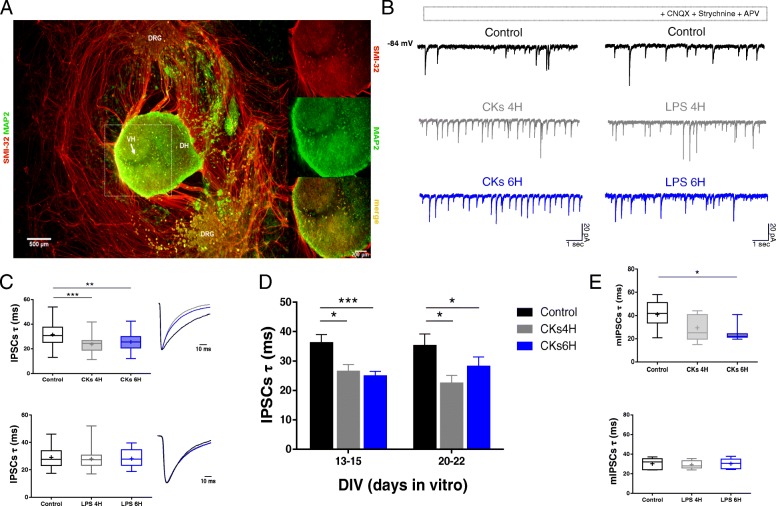
Cytokines affect GABAergic synaptic current kinetics in organotypic spinal culture. **a** Low magnification micrograph of immunofluorescence labeling of organotypic spinal cord slice (14 DIV), SMI-32 (in red), and MAP 2 (in green) markers identify axons and mature neurons. The ventral area (localized by the *ventral fissure*, arrow) highlighted by the dashed square is shown at higher magnification in the right panels. VH = ventral horn, DH = dorsal horn, DRG = dorsal root ganglia. **b** Pharmacologically isolated IPSCs recorded from ventral interneurons at 14 DIV. Control current tracings are in black. CKs 4H (in gray) and 6H (in blue) (left) and LPS 4H (in gray) and 6H (in blue) (right) increased IPSC frequency. **c** Box plots summarize the decay time constant (*τ*) values of IPSCs from pooled experiments in CKs (top) or LPS (bottom); the insets show superimposed and scaled IPSC average tracings (same cells as in **b**); note the changes in IPSC duration in CKs (**P < 0.01; ***P < 0.001, one-way ANOVA). **d** Bar plot (mean ± SEM) of IPSCs *τ* values plotted against DIV; bins are at 13–15 DIV: 36.3 ± 2.7 ms control; 26.5 ± 2.2 ms CKs 4H; 24.5 ± 1.6 ms CKs 6H; *n* = 14, 10, 15, respectively; *P = 0.011 control vs CKs 4H; ***P < 0.001 control vs CKs 6H; bins at 20–22 DIV: 37.6 ± 3.6 ms control; 22.5 ± 2.6 ms CKs 4H; 26.2 ± 2.9 ms CKs 6H; *n* = 8, 6, 6, respectively; *P = 0.011 control vs CKs 4H; *P = 0.041 control vs CKs 6H (one-way ANOVA). **e** Box plots summarize the *τ* values of mIPSCs from pooled experiments in CKs (top) or LPS (bottom), note the changes in mIPSC duration in CKs. *P = 0.024 control vs CKs 6H, one-way ANOVA

We used organotypic spinal cord and DRG co-cultures (Fig. 1a) to study the impact of neuroinflammation on the GABAergic synaptic transmission within ventral horn pre-motor circuits [16]. Two different danger signals were used to trigger neuroinflammation in cultured slices: a pro-inflammatory cocktail of CKs [17] and LPS [19]. In both conditions, after 4H and 6H treatments (see the “Methods” section), patched clamped ventral interneurons displayed a significant increase in the frequency of spontaneous PSCs (represented by heterogeneous inward currents of variable amplitudes, see Additional file 2: Figure S1 A–D) in accordance with previous reports [5, 17, 30, 31].

Since fast Cl^−^-mediated neurotransmission is a potential pro-inflammatory cytokine target in spinal circuits [5, 17, 32], we recorded GABA_A_ receptor-mediated synaptic events (IPSCs; Fig. 1b) pharmacologically isolated in the presence of CNQX (10 μM), APV (25 μM), and strychnine (1 μM), to block AMPA, NMDA, and glycine receptor-mediated synaptic currents [17]. CK and LPS treatments increased IPSC frequency, without altering IPSC amplitudes (sample tracings in Fig. 1b and box plots in Additional file 3: Figure S2 A and B), when compared to control.

We next explored the kinetic properties of the IPSCs. Consistent with our preliminary findings [17], CK treatments (4H and 6H, *n* = 33 and 37, respectively) significantly accelerated the IPSC decay time constant (*τ*) (23.7 ± 6.5 ms CKs 4H; 25.7 ± 6.6 ms CKs 6H; Fig. 1c, top, box plot and scaled averaged IPSCs are superimposed in the inset) when compared to control (31.5 ± 9.6 ms control, *n* = 40; ***P < 0.001 control vs CKs 4H, **P = 0.004 control vs CKs 6H; Fig. 1c). Conversely, LPS (4H and 6H, *n* = 34 and 28, respectively) left the *τ* of the IPSCs unchanged (27.9 ± 7.1 ms LPS 4H; 28.1 ± 6.3 ms LPS 6H) in respect to control (29.1 ± 7.4 ms control, *n* = 37; Fig. 1c, bottom box plot and scaled averaged IPSCs are superimposed in the inset). The IPSC rise-time values (Additional file 3: Figure S2 C) were unaffected by all treatments. Since IPSC decay time may be developmentally regulated [32], we plotted the *τ* values detected in control and in CKs 4H and 6H against two time of growth in vitro. Bar plots in Fig. 1d show that CKs shortened the GABAergic current duration at any age of maturation in vitro, thus excluding a correlation between the CK modulation of IPSC decay time and the developmental stage of spinal cord slices in vitro.

We extended our characterization to the properties of miniature GABAergic currents (mPSCs; recorded in the presence of TTX). The results in this group of cells (Fig. 1e) confirmed that CKs affected mPSC decay kinetics (mPSCs *τ* value 40.9 ± 11.7 ms in control; 29.4 ± 11.6 ms in CKs 4H; 24.3 ± 6.4 ms in CKs 6H; *n* = 9, 7, 9, respectively; *P = 0.024 control vs CKs 6H) with a trend similar to that of spontaneous IPSCs. mIPSC decay time remained unchanged upon LPS treatments (30.2 ± 5.9 ms control; 29.3 ± 4.3 ms LPS 4H; 30.2 ± 5.3 ms LPS 6H; *n* = 5, 5, 5, respectively; Fig. 1e, bottom) in accordance with the spontaneous IPSCs.

We next examined by immunofluorescence the presence and distribution of GABAergic neurons in spinal ventral horns, targeting either isoforms of GABA-synthesizing enzyme (glutamate decarboxylase, GAD), namely GAD65/67 [33]. We quantified and compared GAD65/67 labeling in all conditions (Additional file 4: Figure S3, A–D). Immunoreactivity for GAD65/67 was visible throughout the spinal explants where neurons were visualized using a specific marker (class III β-tubulin, Additional file 4: Figure S3 A and C). At lower magnification, scattered soma, extensive neural processes, and bouton-like structures (named clusters, see the “Methods” section) appeared to be stained for both GAD isoforms and were not affected by CK or LPS treatments, quantified in Additional file 4: Figure S3 B and D.

### Resident neuroglia reactivity to CKs and LPS in spinal organotypic slices

**Fig. 2:**
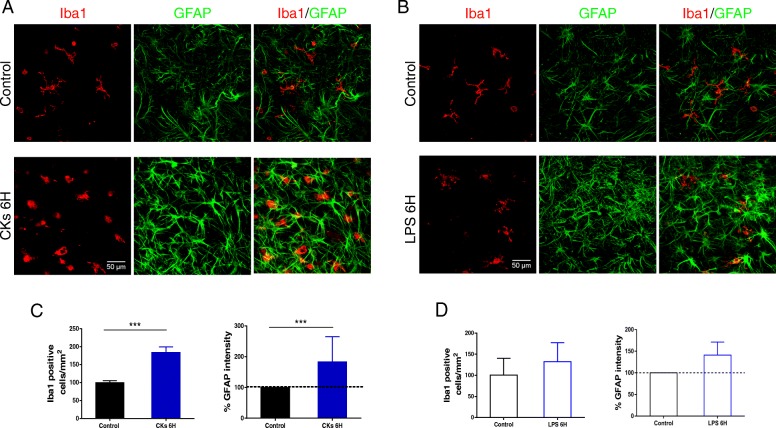
CK and LPS modulation of resident microglia and astrocytes in organotypic spinal cultures. **a**, **b** Representative confocal images of organotypic spinal slices immunolabeled for Iba1 (in red) and GFAP (in green), visualizing microglia and astrocytes, respectively, prior and after CKs (in **a**) or LPS (in **b**) treatments (4H and 6H). **c**, **d** Bar plots summarize Iba1^+^ cells/mm^2^ and GFAP intensity (expressed as percentage of control) prior and after CKs (6H; in **c**) or LPS (6H; in **d**) treatments. Bar plots (mean ± SEM); ***P < 0.001, one-way ANOVA

In response to different microenvironment stimuli, microglia and astrocytes may switch to active states, highlighted by changes in cell function, number, and/or morphology. Microglia and astrocytes were visualized in organotypic spinal explants by Iba1 and GFAP co-immunolabeling, shown in Fig. 2 a and b.

CKs 4H and 6H promoted a significant increase in Iba1^+^ cells (99.8 ± 5.5 cells/mm^2^ control; 155.3 ± 21.5 cells/mm^2^ CKs 4H; 183.7 ± 15.7 cells/mm^2^ CKs 6H; *P = 0.032 control vs CKs 4H; ***P < 0.001 control vs CKs 6H; summarized in Additional file 1: Table S1 for 4H and in Fig. 2c, left, for 6H). The same treatments promptly induced a significant increase in GFAP intensity (158.1 ± 35.7% CKs 4H; 229.3 ± 37.3% CKs 6H; *P = 0.024 control vs CKs 4H, ***P < 0.001 vs CKs 6H; ***P < 0.001 CKs 4H vs CKs 6H; summarized in Additional file 1: Table S1 for 4H and Fig. 2c, right, for 6H). Differently, LPS, summarized for 4H in Additional file 1: Table S1 and in the plots in Fig. 2d for 6H, provoked only mild increases in Iba1^+^ cells (100.8 ± 8.2 cell/mm^2^ control; 112.8 ± 7.4 cell/mm^2^ LPS 4H; 132.2 ± 11.7 cell/mm^2^ LPS 6H) and in the GFAP intensity (110.4 ± 18.7% LPS 4H; 141.1 ± 15.1% LPS 6H).

**Fig. 3:**
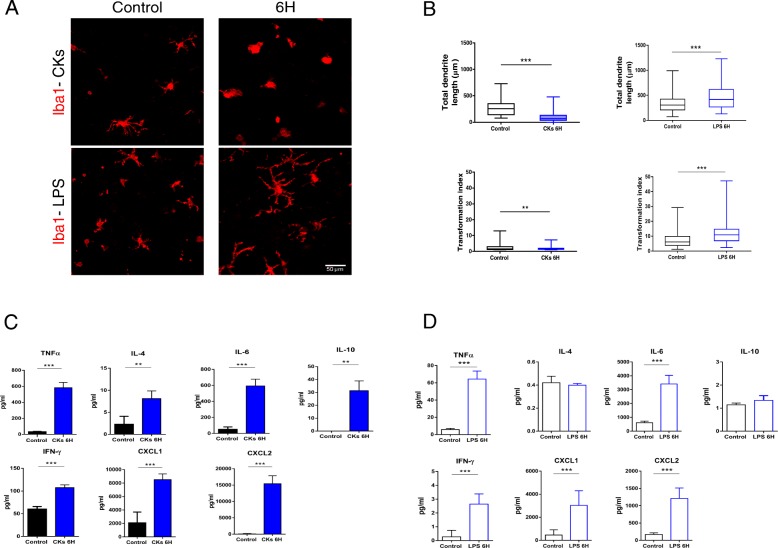
CKs and LPS induce opposite changes in microglia morphology. **a** Representative confocal images comparing Iba1 immunolabeling in untreated (control) and CK- and LPS-treated (4H and 6H) cultures. **b** Box plots show pooled data from different experiments. Note the significant decrease in total dendrites length and transformation indices upon CKs 6H (left); on the opposite, a significant increase in total dendrites length and in the transformation indices is reported at LPS 6H (right). **P < 0.01, ***P < 0.001, Kruskal-Wallis test (Dunn’s post hoc test). **c**, **d** Production of cytokines (TNF-α; IL-4; IL-6; IL-10; INF-γ) and chemokines (CXCL1; CXCL2) determined by Milliplex assay of organotypic culture supernatants after incubation with CKs (**c**) and LPS (**d**) for 6H. Bar plot (mean ± SEM of 22 and 14 slices, respectively); **P < 0.01, ***P < 0.001, one-way ANOVA

We further assessed CK and LPS ability to shape the morphology of Iba1^+^ microglia at 6H. To this aim, we quantified the total dendrite length [34] together with the transformation index (results summarized in Fig. 3a, b; [25]). Upon CKs 6H treatments, microglia showed a significant decrease in both the total dendrite lengths (270.3 ± 144.8 μm control; 93.8 ± 81.2 μm CKs 6H; ***P < 0.001 control vs CKs 6H) and the transformation index (2.7 ± 2.4 control; 1.7 ± 0.7 CKs 6H; **P < 0.005 control vs CKs 6H). In parallel, in slices stimulated by LPS, we observed, at 6H, changes in microglia morphology that seem to indicate a different stage of activation [35]. In fact, Fig. 3 a and b show a significant increment of the Iba1^+^ cell dendrite length at LPS 6H (346.6 ± 194.9 μm control; 472.6 ± 255.9 μm LPS 6H; ***P < 0.001 control vs LPS 6H) and of the transformation index (7.2 ± 5.3 control; 12.2 ± 7.7 LPS 6H; ***P < 0.001 control vs LPS 6H). Finally, we evaluated and compared the production of cytokines and chemokines by spinal slices in response to pro-inflammatory stress at 6H (for CKs *n* = 14 slices and for LPS *n* = 22 slices, from 3 different culture series). The summarizing plots of Fig. 3c, d show that the exposure to CKs (c) and LPS (d) significantly increased, although to a different extent, the release of pro-inflammatory cytokines, measured in the supernatant, such as TNF-α, IL-6, and INF-γ, as well as the release of chemokines including CXCL1 and CXCL2 necessary for the recruitment of innate immune cells. Interestingly, IL-4 and IL-10 are significantly raised upon CK stimuli, but are not changed after LPS treatments. The different pro- and anti-inflammatory cytokine network, in addition to the production of chemokines, suggests the induction of alternative activation mechanisms in spinal slices stimulated by CKs and LPS at the time point (6H) analyzed.

**Fig. 4:**
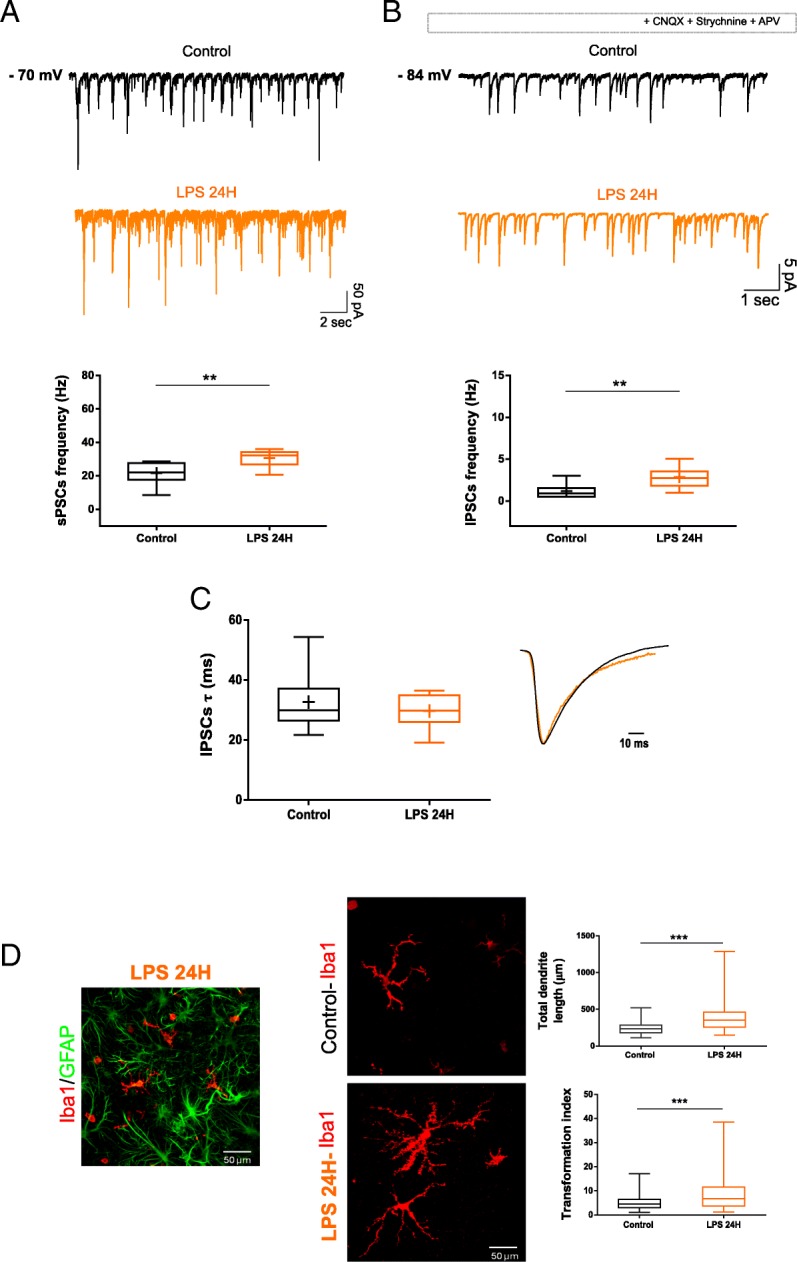
Long-term exposure to LPS in organotypic spinal slices is not altering GABAergic currents. **a** Representative traces of spontaneous PSCs recorded from control (in black) and LPS 24H (in orange) ventral interneurons. The box plot shows a significant increase in PSC frequency upon LPS 24H. **P = 0.003, Mann-Whitney test. **b** Pharmacologically isolated IPSCs are recorded from control (in black) and LPS 24H (in orange) ventral interneuron (same cells as in **a**). The box plot summarizes the IPSC frequency from pooled experiments and exhibits a significantly increased frequency brought about by LPS 24H. **P = 0.007, Mann-Whitney test. **c** The box plot summarizes the values of *τ* for control and LPS; in the inset, averaged scaled and superimposed traces of the two conditions are shown. Note that no changes were detected in IPSC decay time constant. **d** Left, representative image of the LPS 24H-treated organotypic culture labeled with GFAP (green) and Iba1 (red). Right, high magnification Iba1 micrographs are shown (middle), and plots summarize the morphology changes of microglia upon LPS 24H. Note the significant increase in dendrites length (top) and in the transformation index (bottom); ***P < 0.001, Mann-Whitney test

In the central nervous system, LPS binds to the Toll-like receptors (TLRs), especially TLR4, expressed on the microglia surface. This signal involves several proteins resulting in the production and release of cytokines, chemokines, and other inflammatory factors [36]. Since LPS, differently from CKs [37], may not act directly on neurons, we tested whether a longer (24H) exposure to LPS may ultimately lead to changes in GABAergic transmission and kinetic. Figure 4 reports the effects of LPS 24H in terms of PSC frequency (21.7 ± 6.7 Hz control; 30.5 ± 5.0 Hz LPS 24H; *n* = 8 and 12, respectively; **P = 0.003 control vs LPS 24H; Fig. 4a), IPSC frequency (1.2 ± 0.8 Hz control; 2.5 ± 0.9 Hz LPS 24H; **P = 0.007 control vs LPS 24H; Fig. 4b), and IPSC decay time constant (32.7 ± 10.2 ms control; 29.7 ± 5.5 ms LPS 24H, Fig. 4c, scaled averaged IPSCs are superimposed in the inset). In addition, Iba1^+^ cells at LPS 24H (Fig. 4 D) displayed the characteristic morphology with longer branching in ramified cells (quantified in plots of Fig. 4d, total dendrites length: 249.6 ± 96.0 μm control; 393.4 ± 216.9 μm LPS 24H; ***P < 0.001 control vs LPS 24H; transformation indices: 5.2 ± 3.1 control; 8.6 ± 7.1 LPS 24H). These results indicated that the kinetic of GABAergic currents was not modulated by longer incubation in this danger signal, and the effects on microglia dendrite lengths stabilized after 6H.

### CKs modulate GABAergic current duration through changes in GABA_A_R subunits composition

We further explored the mechanisms responsible for IPSC shortening due to CKs. The decay of IPSCs is also faster, upon CK treatments, in unitary synaptic events (mIPSCs), ruling out the involvement of presynaptic processes affecting IPSC time course, such as neurotransmitter release synchronization. Moreover, the absence of changes in IPSC rise time (Additional file 3: Figure S2 C) suggests that differences in recording conditions, location of synapses or electronic filtering, are unlikely to have affected our observations.

**Fig. 5:**
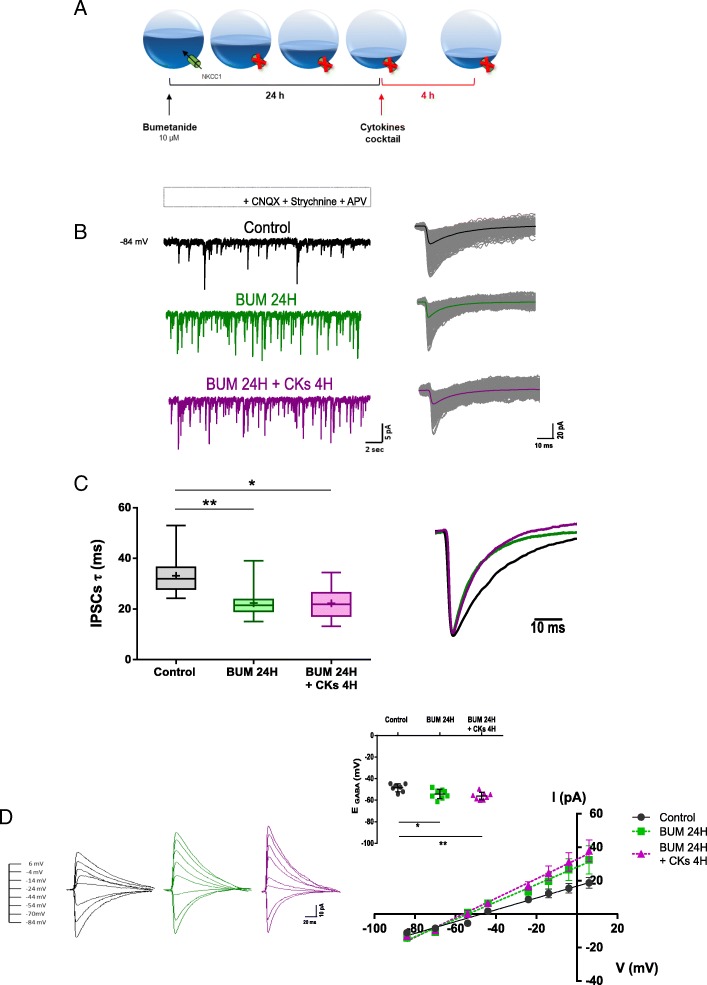
Effects of CKs on GABAergic synaptic current duration in the presence of bumetanide. **a** Sketch of the protocol used to treat slices with bumetanide for 24H (BUM 24H) followed by CKs 4H. **b** Representative traces (left) and superimposed isolated events (right) of IPSCs in control, after BUM 24H (green), and BUM 24H + CKs 4H (magenta). **c** The box plot summarizes the IPSCs *τ* values measured in the three conditions; note the significant reduction in IPSCs *τ* in BUM 24H and BUM 24H + CKs 4H, in respect to control. Inset: superimposed averaged and scaled IPSCs in the three conditions. *P = 0.012, **P = 0.005, Kruskal-Wallis test. **d** Top, IPSCs averaged and superimposed traces recorded at different V_h_ in control (black), BUM 24H (green), and BUM 24H + CKs 4H (magenta). Bottom, I/V curves obtained by plotting GABA_A_-IPSCs mean amplitude against V_h_. Note in the inset the significant differences in the estimated IPSCs reversal potential (E_GABA_) at BUM 24H and BUM 24H + CKs 4H, when compared to control. *P < 0.05, **P < 0.01, one-way ANOVA

Differences in the intracellular chloride concentration [Cl^−^]_i_ were reported to affect IPSC kinetics [38, 39]. We incubated organotypic slices with bumetanide (10 μM; 24H, *n* = 11), a blocker of NKCC1 activity [24], a membrane-protein described as the most abundant co-transporter determining intracellular chloride levels [40], to experimentally reduce [Cl^−^]_i_ prior to CKs 4H (*n* = 10; see sketch of the experimental settings in Fig. 5a).

Bumetanide per se induced an increase in PSC and IPSC frequencies that were slightly, although not significantly, further improved by CKs 4H (Fig. 5b for sample tracings and Additional file 5: Figure S4). More intriguingly, bumetanide reduced significantly the duration of GABAergic currents (*τ* = 33.1 ± 8.1 ms control; *τ* = 22.3 ± 6.2 ms BUM 24H; **P = 0.005 control vs BUM 24H) that were not further shortened by CKs 4H (*τ* = 22.3 ± 6.5 ms BUM 24H + CKs 4H; *P = 0.012 control vs BUM 24H + CKs 4H; Fig. 5c).

Figure 5 d shows the measurement of the reversal potential of IPSCs in control, BUM 24H, and BUM 24H + CKs 4H. The estimated *E*_Cl_ value in control (− 48.5 ± 3.7 mV; *n* = 7) was close to the approximate theoretical value expected for the Cl^−^ equilibrium potential for our intracellular and extracellular chloride concentrations (− 50 mV; [26]). However, the reversal potential was significantly shifted to more negative values by blocking NKCC1 (− 54.2 ± 4.3 mV BUM 24H, *n* = 8; *P = 0.025 control vs BUM 24H), suggesting that local intracellular chloride concentrations are lower (estimated from 24 to 19 mM). It is important to note that in organotypic cultures, upon bumetanide treatments, the Cl^−^ reversal potential differed from the predicted theoretical value, suggesting a real shift in the internal chloride concentration as a result of reduced co-transport [41, 42], regardless the 24 mM Cl^−^ intracellular pipette solution [17].

Pro-inflammatory CKs, in the presence of NKCC1 block, slightly increased such a shift only when compared to controls (− 56.2 ± 3.5 mV BUM 24H + CKs 4H; *n* = 8; **P = 0.003 control vs BUM 24H + CKs 4H). The absence of significant changes in IPSC decay time constant and reversal once CKs were incubated in the presence of bumetanide might indicate an occluding mechanisms between CKs and bumetanide in regulating [Cl^−^]_i_.

To shed light in CK potential regulation of intracellular chloride, and thus of IPSCs *τ*, we estimated and compared *E*_Cl_ in control, CKs, and LPS treatments. Additional file 6: Figure S5 shows that the reversal potential of IPSCs was not altered by these treatments alone (− 52.0 ± 7.5 mV control; − 51.5 ± 5.2 mV CKs 4H; *n* = 9, 10, respectively; − 49.6 ± 7.8 mV control; − 49.7 ± 9.9 mV LPS 4H; *n* = 13, 8, respectively). Regardless of the similar *E*_Cl_ extrapolated in all the recording conditions, only CKs 4H induced the expected changes in the IPSC duration (*τ* = 31.8 ± 5.1 ms control; *τ* = 23.9 ± 8.1 ms CKs 4H; *P = 0.031 control vs CKs 4H; Student *t* test; *τ* = 26.4 ± 5.2 ms control; *τ* = 26.2 ± 6.3 ms LPS 4H; respectively). These results show that changes in *E*_Cl_ might indeed modulate IPSC duration in the organotypic spinal interneurons; however, CKs apparently are not tuning the inhibitory current duration by shifts in the *E*_Cl_.

**Fig. 6:**
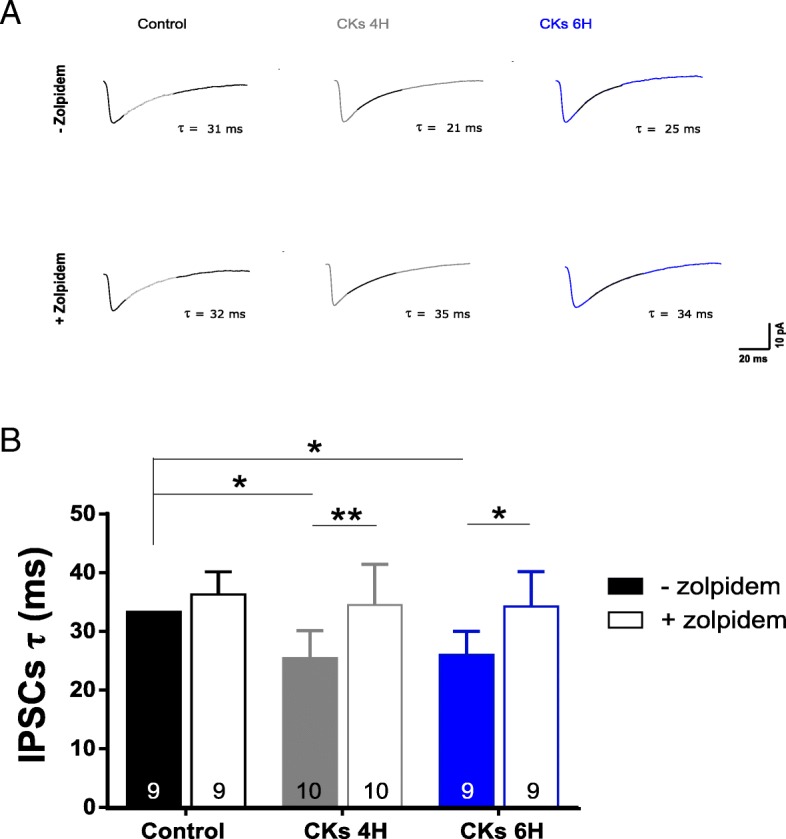
Zolpidem effects on GABAergic synaptic current duration before and after CK treatments. **a** Sample tracings of averaged IPSCs recorded in control (black) and CKs (4H in gray and 6H in blue) in the presence (+) or in the absence (−) of zolpidem. Note the superimposed fitting used to calculate *τ* values. **b** Bar plot summarizes the *τ* values of IPSCs in control, CKs 4H, and CKs 6H, before and after bath application of zolpidem. Note that the significant decrease in IPSCs τ upon CKs treatments (4H and 6H) was reversed by zolpidem. *P < 0.05, **P < 0.01, one-way ANOVA

A well-documented post-synaptic process that changes GABAergic inhibition is the switch in the α1-subunit expression reported to modulate IPSC kinetics, which become faster [43]. To address the potential changes in the receptor subunit composition due to CK treatment, we tested IPSC kinetics in the presence of zolpidem (100 nM; 15–20 min), an allosteric modulator of GABA_A_R subunits that at low concentrations is highly selective for the GABA_A_R α1 subunit [44]. Figure 6a shows sample superimposed isolated IPSCs recorded from control, CKs 4H, and CKs 6H, before and after zolpidem applications. Also, in this set of recordings, after CKs 4H and 6H, IPSCs *τ* was significantly reduced (33.3 ± 4.5 ms control; 25.4 ± 4.7 ms CKs 4H; 26.0 ± 4.0 ms CKs 6H; *n* = 9, 10, 9, respectively; *P = 0.019 control vs CKs 4H; *P = 0.045 control vs CKs 6H). Subsequent applications of zolpidem did not alter IPSCs *τ* in control (36.3 ± 3.9 ms), while significantly prolonged *τ* values detected in CKs 4H and 6H (34.5 ± 6.9 ms CKs 4H; 34.2 ± 6.0 ms CKs 6H; **P = 0.003 CKs 4H − Zolpidem vs CKs 4H + zolpidem; *P = 0.017 CKs 6H -zolpidem vs CKs 6H + zolpidem), which did not differ anymore from control IPSCs (summarized in the bar plots of Fig. 6 B). These results strongly suggest that CK treatments regulated the duration of GABAergic inhibition via post-synaptic changes of the α1 subunit.

## Discussion

Our study targets synaptic changes in response to local CNS tissue reactivity, experimentally induced by CKs or LPS and involving resident neuroglia. In particular, we used the spinal organotypic cultures to focus on the interplay between local inflammation and the dynamics of GABAergic currents, whose altered decay may represent a subtle alteration able to trigger neuronal network dysfunction, potentially involved in neurodegenerative processes [45–47]. The organotypic slice model represents a high-density cell system, where the 3D-architecture of specific resident cells, neuronal and non-neuronal, is preserved in a tissue culture setting [15–17, 48]. The main finding of the current work is that local inflammation in organotypic spinal slices induced by CKs and LPS stimulations boosts network activity already after 4H treatments, as shown by the augmented PSCs and IPSCs frequency; however, only tissue reactivity brought about by CKs specifically reduced GABAergic current duration. We adopted short-term treatments to trigger inflammatory responses, without affecting neuronal membrane properties or inducing direct neurotoxicity, yet still able to alter synaptic transmission [17, 49].

The ability of CKs, directly, or of LPS-activated immune cells, indirectly, to increase synaptic activity or neuronal excitability has been previously described using several experimental settings and in different CNS structures [5, 17, 30, 31, 50, 51]. Yet, mechanistically, the influence on synaptic function of acute or prolonged exposures to inflammatory milieus has led often to controversial results, involving the glutamatergic signaling system [6, 52, 53], or the inhibitory synaptic transmission [5, 30, 54], as well as neuronal excitability [31, 50, 55], clearly indicating the complexity of the signaling pathways activated upon inflammation. In addition, the output readout used in these studies, i.e., post-synaptic currents, might be misleading, due to the variable amount of homeostatic plasticity taking place once destabilizing influences alter synaptic transmission [56]. Regardless the mechanisms leading to improved synaptic activity, only the direct exposure to CKs regulated the GABAergic current decay, probably via a post-synaptic mechanisms, as indicated by the detected changes in mIPSC’s *τ* [26, 57]. The absence of LPS ability, even upon prolonged exposure of the slices, to regulate the inhibitory current decay was apparently not due to a lack of LPS activation of inflammation, a notion supported by the LPS-mediated increase in synaptic activity and by the cytokines and chemokines produced by LPS-treated slices, despite the low GFAP-positive cell reactivity and the changes in microglia morphology detected by immunocytochemistry, different to those reported in CKs. To note, the morphology of resident neuroglia together with the small increase in GFAP expression upon LPS treatment may indicate a different state of activation, supported by a different profile of CK network and production, providing an alternative (i.e., to CKs), inflammation-mediated regulation of brain functions [35, 58]. LPS is experimentally used to mimic CNS bacterial inflammation [59], whereas CKs mimic the inflammatory network present when immune system intervenes in the CNS. These two functional conditions may affect synapses differently, and future investigations may allow elucidating CNS pathological conditions related to distinct etiologies. Indeed, we have to consider the diverse mechanisms of action triggered by CKs and LPS [60–63] which both act through precise receptors on microglia and neurons, affecting the microglia/neuron communication and function [64].

CK regulation of GABAergic current in the absence of changes in GABA synthesis is thus a specific feature of the selected CK cocktail, able to mimic an inflammatory reaction involved in neuropathy [2, 6, 17], and the mechanisms of this modulation might be a targetable pathway in spinal neuroinflammatory disease treatments.

We examined the main variables that might conceivably affect the kinetic properties of GABA_A_R and therefore the GABAergic-PSC time course. First, we excluded the possibility of differences in the intracellular chloride concentration, brought about by neuroinflammation [38, 65] that could affect IPSC kinetics [66], as confirmed by our experiments where NKCC1 was pharmacologically blocked, leading to a shift in Cl^−^ reversal potential. Our measurements show that the Cl^−^ reversal potential was similar in the cultures treated by CKs or LPS, in the absence of bumetanide, yet CKs still induced a significant shortening of the GABAergic current duration. In bumetanide-treated cultures, the Cl^−^ reversal potential differed from the predicted theoretical value. In our recording conditions, it is not possible to confirm a real shift in the internal chloride concentration as a result of the decreased influx [41, 42]. However, the detected shifts in bumetanide are consistent with a 19 mM Cl^−^ intracellular concentration.

An alternative process that changes IPSC current duration in the CNS is the GABA_A_R α-subunit composition [39, 67–69]. In particular, α1-subunit is responsible for fast deactivation, which results in faster-decaying currents [39, 69]. Our hypothesis, of an increased expression of α1-subunit upon CKs treatment, was supported by the efficacy of zolpidem [44] to prolong IPSC duration only in CK-treated slices.

Our results describe for the first time a selective mechanism that could be triggered during inflammatory stress. In particular, under pathological conditions, the switch of GABA_A_R α1-subunit would induce faster GABA_A_R decay time, weakening the IPSCs transmission. Therefore, lower IPSC duration could contribute to providing an aberrant excitatory transmission critical for pre-motor circuit tasks.

## Conclusions

We exploited spinal cord explant cultures to investigate two diverse immune conditions in the CNS, characterized by different inflammatory networks and products, thus providing alternative inflammation-mediated regulation of the CNS functions. We have shown that these two functional conditions affect inhibitory synapses differently, and we hypothesized that the mechanisms of this modulation might be a targetable pathway in spinal neuroinflammatory disease treatments.

## Additional files

Additional file 1: **Table S1.** Neuroglial cell reactivity upon 4-h treatments in CKs and LPS. (PDF 1613 kb)

Additional file 2: ** Figure S1.** CKs and LPS increase sPSC frequency in organotypic slices A-B, Representative current tracings of sPSCs recorded in control (black) and after incubation in CKs (4H in gray and 6H in blue; left) or in LPS (4H in gray and 6H in blue; right). C-D, Box plots summarize the increase in sPSC frequency (20.3 ± 9.5 Hz control; 27.9 ± 9.4 Hz CKs 4H; 28.8 ± 9.4 Hz CKs 6H; ***P < 0.001 control vs CKs 4H and control vs CKs 6H, one-way ANOVA) and in LPS (21.4 ± 9.7 control; 28.4 ± 10.4 LPS 4H; 33.3 ± 9.8 LPS 6H; **P = 0.008 control vs LPS 4H; ***P < 0.001 control vs LPS 6H, one-way ANOVA) treatments. (PDF 37 kb)

Additional file 3: ** Figure S2.** CKs and LPS increase IPSC frequency in organotypic slices A–C. Box plots illustrate the mean value of IPSCs frequency (A), amplitude (B), and rise time (C) upon CK and LPS treatments. A significant increase was observed in the IPSC frequency at CKs 4H and 6H, when compared to control (3.3 ± 1.5 Hz control; 4.7 ± 1.9 Hz CKs 4H; 4.6 ± 2.0 Hz CKs 6H; *n* = 40, 33, 37, respectively; **P = 0.003 control vs CKs 4H and **P = 0.006 vs CKs 6H, one-way ANOVA) and at LPS 4H and 6H when compared to their relative control (1.7 ± 0.9 Hz control; 3.1 ± 2.6 Hz LPS 4H; 3.4 ± 2.7 Hz LPS 6H; *n* = 34, 33, 27, respectively; *P = 0.002, control vs LPS 4H, and **P = 0.001 vs LPS 6H, one-way ANOVA). IPSC amplitude (17.5 ± 10.5 pA control; 19.4 ± 9.1 pA CKs 4H; 17.0 ± 11.2 pA CKs 6H; 15.7 ± 8.9 pA control; 11.3 ± 4.5 pA LPS 4H; 13.1 ± 7.3 pA LPS 6H) and rise time (2.5 ± 0.8 ms control; 2.1 ± 0.8 ms CKs 4H; 2.6 ± 1.4 ms CKs 6H; 2.6 ± 1.0 ms control; 2.5 ± 1.0 ms LPS 4H; 2.6 ± 0.9 ms LPS 6H) were unaffected by CK or LPS treatments. (PDF 6885 kb)

Additional file 4: ** Figure S3.** GAD65/67 immunoreactivity in organotypic slices before and after CK or LPS treatments A and C. Representative images of spinal slices labeled for β-tubulin III (in blue) and GAD65/67 (in red) show GABAergic neurons in untreated (control) and CK- and LPS-treated (4H and 6H) ventral area of spinal organotypic slices (14 DIV) B and D. Bar plots summarize the normalized GAD65/67 clusters (1483 ± 62.2 control; 1503 ± 49.9 CKs 4H; 1403 ± 27.2 CKs 6H; 1295 ± 45.3 control; 1300 ± 46.3 LPS 4H; 1382 ± 54.4 LPS 6H) and the GAD65/67 intensity in a.u. (379.1 ± 29.4 control; 423.9 ± 40.8 CKs 4H; 383.9 ± 38.5 CKs 6H; 417.3 ± 46.9 control; 391.3 ± 58.4 LPS 4H; 433.3 ± 46.5 LPS 6H). (PDF 28 kb)

Additional file 5: ** Figure S4 **Spontaneous PSC and IPSC frequency in bumetanide 24 h before and after CKs 4H. **A** Left, box plots of PSCs frequency values from control, bumetanide-treated slices prior and after CKs. Note the significant increase in PSC frequency BUM 24H and BUM 24H + CKs 4H compared to control (24.4 ± 7.8 Hz control; 37.1 ± 11.4 Hz BUM 24H; 42.6 ± 11.2 Hz BUM 24H + CKs 4H; *n* = 9, 10, 9, respectively; *P = 0.033 control vs BUM 24H; **P = 0.003 control vs BUM 24H + CKs 4H, one-way ANOVA). Right, box plots of IPSCs frequency values upon BUM 24H and BUM 24H + CKs 4H compared to control (3.1 ± 1.4 Hz control; 6.0 ± 2.8 Hz BUM 24H; 6.6 ± 2.5 Hz BUM 24H + CKs 4H; *n* = 10, 11, 10, respectively; *P = 0.019 control vs BUM 24H; **P = 0.005 control vs BUM 24H + CKs 4H, one-way ANOVA). (PDF 278 kb)

Additional file 6: ** Figure S5. **E_GABA_ estimated in control, CKs 4H and LPS 4H. IPSCs averaged and superimposed traces (top) recorded at different V_h_ in control, CKs 4H, and LPS 4H. Bottom, I/V curves were obtained by plotting GABA_A_-PSCs mean amplitude against Vh. Inset, note the similar (E_GABA_) in all conditions. *P < 0.05, **P < 0.01, one-way ANOVA. (PDF 13 kb)

## Data Availability

The datasets supporting the conclusion of this article are included within the article (and its additional files). The datasets generated and/or analyzed during the current study are stored in a public repository and are available from the corresponding authors on reasonable request.

## Abbreviations

APV: (2*R*)-amino-5-phosphonovaleric acid
BSA: Bovine serum albumin
BUM: Bumetanide
CKs: Cytokines
CNQX: 6-Cyano-7-nitroquinoxaline-2,3-dione
DH: Dorsal horn
DIV: Days in vitro
DRG: Dorsal root ganglia
FBS: Fetal bovine serum
GAD: Glutamate decarboxylase
GFAP: Glial fibrillary acidic protein
GM-CSF: Granulocyte-macrophage colony-stimulating factor
Iba1: Ionized calcium-binding adapter molecule 1
IL-1β: Interleukin-1beta
LPS: Lipopolysaccharide
MAP 2: Microtubule-associated protein 2
mPSCs: Miniature post-synaptic currents
PBS: Phosphate-buffered saline
PSCs: Post-synaptic currents
RT: Room temperature
SMI-32: Neurofilament H non-phosphorylated
TNF-α: Tumor-necrosis factor-alfa
TTX: Tetrodotoxin
VH: Ventral horn
