# Supplementary figures and images for: Cytokine inflammatory threat, but not LPS one, shortens GABAergic synaptic currents in the mouse spinal cord organotypic cultures
Source: J Neuroinflammation. 2019 Jun 25;16:127. doi: 10.1186/s12974-019-1519-z (PMC6593520; doi:10.1186/s12974-019-1519-z)

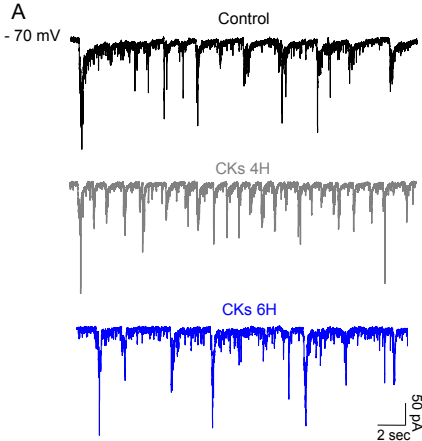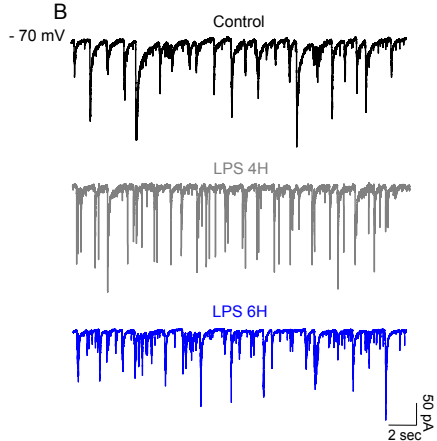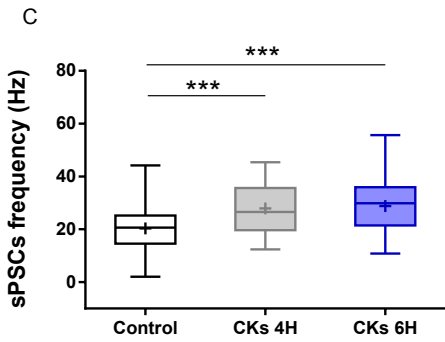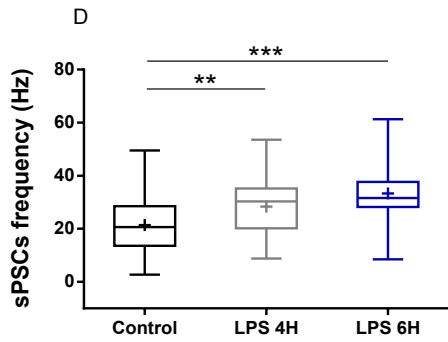

Supplement: Supplementary file 1 — Table S1. Neuroglial cell reactivity upon 4-h treatments in CKs and LPS. (PDF 1613 kb) [file 12974_2019_1519_MOESM1_ESM.pdf]

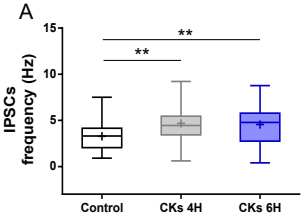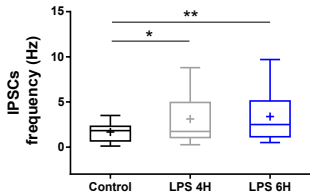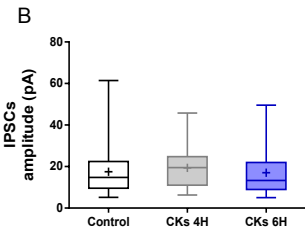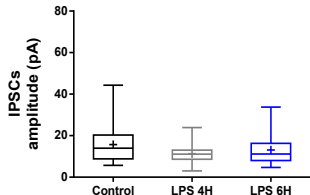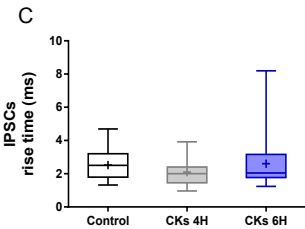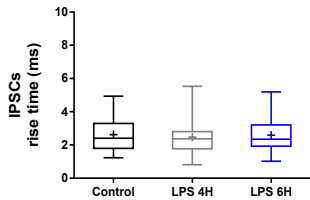

Supplement: Supplementary file 2 — Figure S1. CKs and LPS increase sPSC frequency in organotypic slices A-B, Representative current tracings of sPSCs recorded in control (black) and after incubation in CKs (4H in gray and 6H in blue; left) or in LPS (4H in gray and 6H in blue; right). C-D, Box plots summarize the increase in sPSC frequency (20.3 ± 9.5 Hz control; 27.9 ± 9.4 Hz CKs 4H; 28.8 ± 9.4 Hz CKs 6H; ***P < 0.001 control vs CKs 4H and control vs CKs 6H, one-way ANOVA) and in LPS (21.4 ± 9.7 control; 28.4 ± 10.4 LPS 4H; 33.3 ± 9.8 LPS 6H; **P = 0.008 control vs LPS 4H; ***P < 0.001 control vs LPS 6H, one-way ANOVA) treatments. (PDF 37 kb) [file 12974_2019_1519_MOESM2_ESM.pdf]

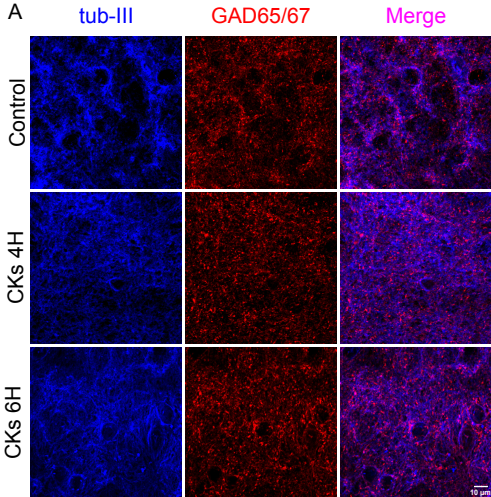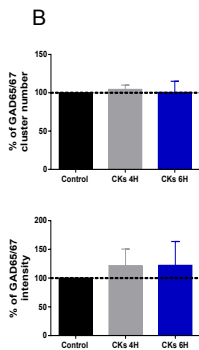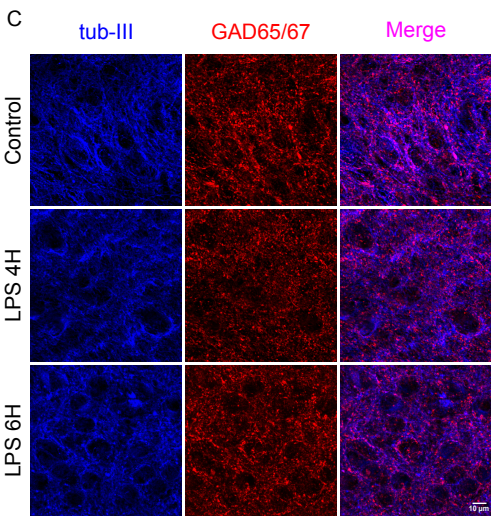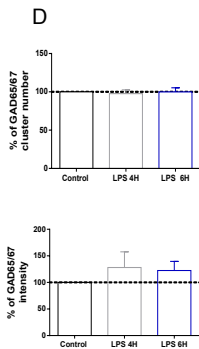

Supplement: Supplementary file 3 — Figure S2. CKs and LPS increase IPSC frequency in organotypic slices A–C. Box plots illustrate the mean value of IPSCs frequency (A), amplitude (B), and rise time (C) upon CK and LPS treatments. A significant increase was observed in the IPSC frequency at CKs 4H and 6H, when compared to control (3.3 ± 1.5 Hz control; 4.7 ± 1.9 Hz CKs 4H; 4.6 ± 2.0 Hz CKs 6H; n = 40, 33, 37, respectively; **P = 0.003 control vs CKs 4H and **P = 0.006 vs CKs 6H, one-way ANOVA) and at LPS 4H and 6H when compared to their relative control (1.7 ± 0.9 Hz control; 3.1 ± 2.6 Hz LPS 4H; 3.4 ± 2.7 Hz LPS 6H; n = 34, 33, 27, respectively; *P = 0.002, control vs LPS 4H, and **P = 0.001 vs LPS 6H, one-way ANOVA). IPSC amplitude (17.5 ± 10.5 pA control; 19.4 ± 9.1 pA CKs 4H; 17.0 ± 11.2 pA CKs 6H; 15.7 ± 8.9 pA control; 11.3 ± 4.5 pA LPS 4H; 13.1 ± 7.3 pA LPS 6H) and rise time (2.5 ± 0.8 ms control; 2.1 ± 0.8 ms CKs 4H; 2.6 ± 1.4 ms CKs 6H; 2.6 ± 1.0 ms control; 2.5 ± 1.0 ms LPS 4H; 2.6 ± 0.9 ms LPS 6H) were unaffected by CK or LPS treatments. (PDF 6885 kb) [file 12974_2019_1519_MOESM3_ESM.pdf]

A

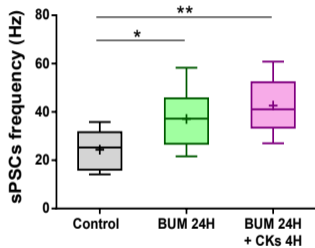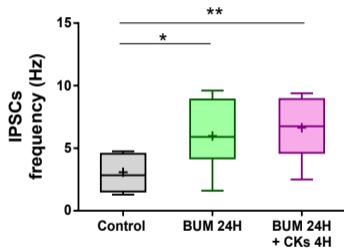

Supplement: Supplementary file 4 — Figure S3. GAD65/67 immunoreactivity in organotypic slices before and after CK or LPS treatments A and C. Representative images of spinal slices labeled for β-tubulin III (in blue) and GAD65/67 (in red) show GABAergic neurons in untreated (control) and CK- and LPS-treated (4H and 6H) ventral area of spinal organotypic slices (14 DIV) B and D. Bar plots summarize the normalized GAD65/67 clusters (1483 ± 62.2 control; 1503 ± 49.9 CKs 4H; 1403 ± 27.2 CKs 6H; 1295 ± 45.3 control; 1300 ± 46.3 LPS 4H; 1382 ± 54.4 LPS 6H) and the GAD65/67 intensity in a.u. (379.1 ± 29.4 control; 423.9 ± 40.8 CKs 4H; 383.9 ± 38.5 CKs 6H; 417.3 ± 46.9 control; 391.3 ± 58.4 LPS 4H; 433.3 ± 46.5 LPS 6H). (PDF 28 kb) [file 12974_2019_1519_MOESM4_ESM.pdf]

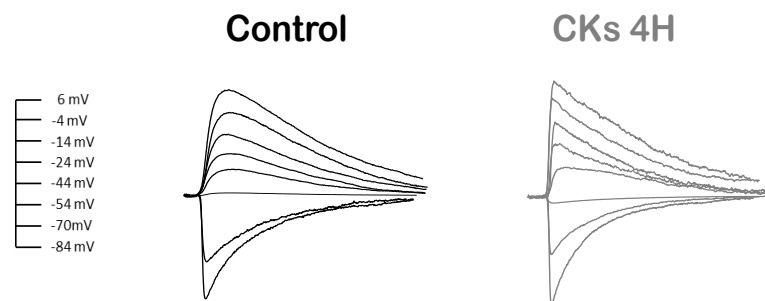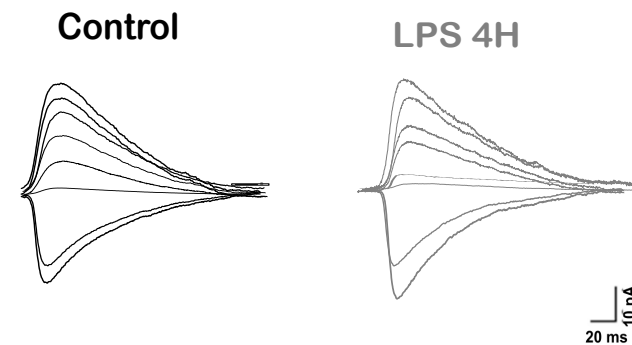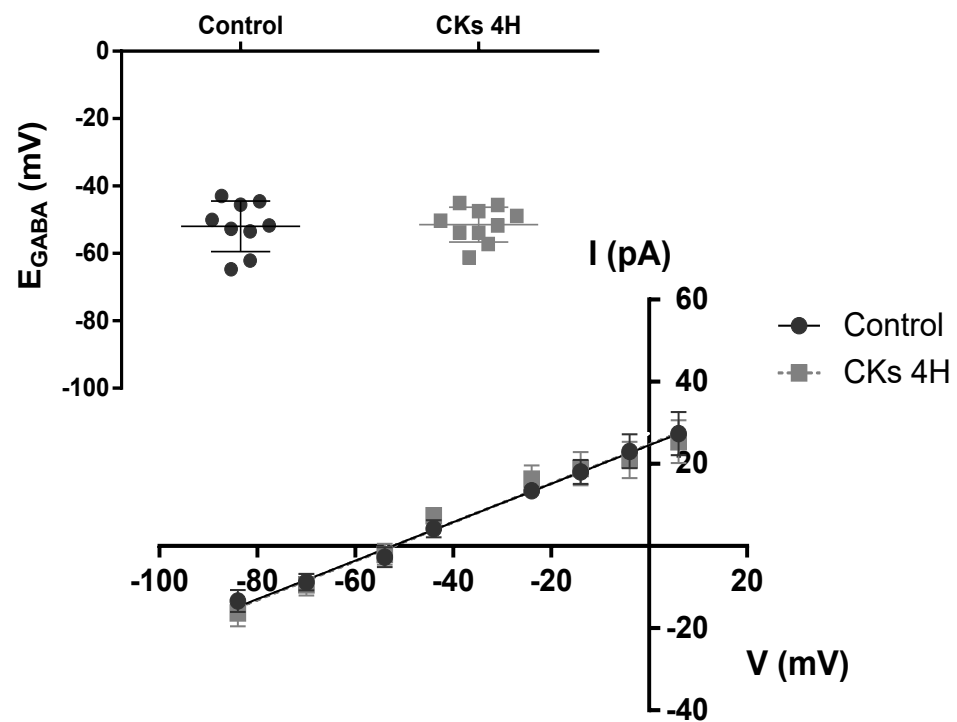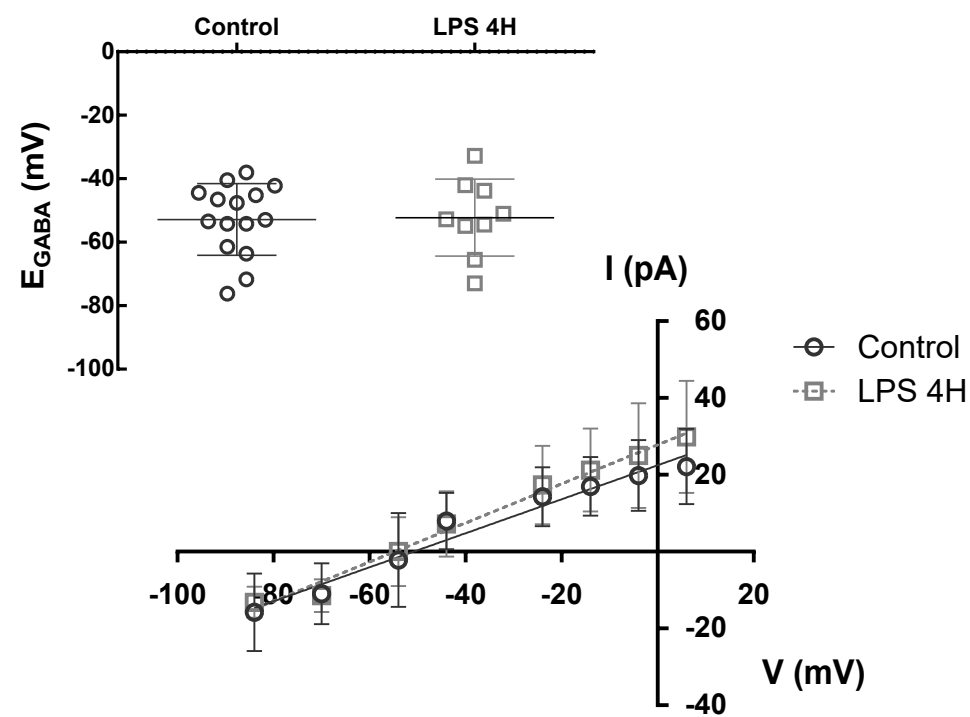

Supplement: Supplementary file 5 — Figure S4 Spontaneous PSC and IPSC frequency in bumetanide 24 h before and after CKs 4H. A Left, box plots of PSCs frequency values from control, bumetanide-treated slices prior and after CKs. Note the significant increase in PSC frequency BUM 24H and BUM 24H + CKs 4H compared to control (24.4 ± 7.8 Hz control; 37.1 ± 11.4 Hz BUM 24H; 42.6 ± 11.2 Hz BUM 24H + CKs 4H; n = 9, 10, 9, respectively; *P = 0.033 control vs BUM 24H; **P = 0.003 control vs BUM 24H + CKs 4H, one-way ANOVA). Right, box plots of IPSCs frequency values upon BUM 24H and BUM 24H + CKs 4H compared to control (3.1 ± 1.4 Hz control; 6.0 ± 2.8 Hz BUM 24H; 6.6 ± 2.5 Hz BUM 24H + CKs 4H; n = 10, 11, 10, respectively; *P = 0.019 control vs BUM 24H; **P = 0.005 control vs BUM 24H + CKs 4H, one-way ANOVA). (PDF 278 kb) [file 12974_2019_1519_MOESM5_ESM.pdf]
