# Supplementary material for: Cytokine inflammatory threat, but not LPS one, shortens GABAergic synaptic currents in the mouse spinal cord organotypic cultures
Source: J Neuroinflammation. 2019 Jun 25;16:127. doi: 10.1186/s12974-019-1519-z (PMC6593520; doi:10.1186/s12974-019-1519-z)
Supplement: Supplementary file 6 — Figure S5. EGABA estimated in control, CKs 4H and LPS 4H. IPSCs averaged and superimposed traces (top) recorded at different Vh in control, CKs 4H, and LPS 4H. Bottom, I/V curves were obtained by plotting GABAA-PSCs mean amplitude against Vh. Inset, note the similar (EGABA) in all conditions. *P < 0.05, **P < 0.01, one-way ANOVA. (PDF 13 kb) [file 12974_2019_1519_MOESM6_ESM.pdf]

Table 1

	CKs 4H	LPS 4H
**Iba1 positive cells (cells/mm^2)**	155.3 ± 21.5	112.8 ± 7.4
	**P* = 0.032 (Control vs CKs 4H)	not significant
**GFAP intensity (%)**	158.1 ± 35.7	110.4 ± 18.7
	**P* = 0.024 (Control vs CKs 4H)	not significant
